# Distributed Particle Filter for Target Tracking: With Reduced Sensor Communications

**DOI:** 10.3390/s16091454

**Published:** 2016-09-09

**Authors:** Tadesse Ghirmai

**Affiliations:** School of STEM, Division of Engineering and Mathematics, University of Washington Bothell, Bothell, WA 98011, USA; tadg@uw.edu; Tel.: +1-425-352-3873

**Keywords:** particle filtering, target-tracking, sensor networks, consensus filter, forward-backward

## Abstract

For efficient and accurate estimation of the location of objects, a network of sensors can be used to detect and track targets in a distributed manner. In nonlinear and/or non-Gaussian dynamic models, distributed particle filtering methods are commonly applied to develop target tracking algorithms. An important consideration in developing a distributed particle filtering algorithm in wireless sensor networks is reducing the size of data exchanged among the sensors because of power and bandwidth constraints. In this paper, we propose a distributed particle filtering algorithm with the objective of reducing the overhead data that is communicated among the sensors. In our algorithm, the sensors exchange information to collaboratively compute the global likelihood function that encompasses the contribution of the measurements towards building the global posterior density of the unknown location parameters. Each sensor, using its own measurement, computes its local likelihood function and approximates it using a Gaussian function. The sensors then propagate only the mean and the covariance of their approximated likelihood functions to other sensors, reducing the communication overhead. The global likelihood function is computed collaboratively from the parameters of the local likelihood functions using an average consensus filter or a forward-backward propagation information exchange strategy.

## 1. Introduction

A network of large number of sensors can be deployed to collect and process data to monitor a certain event. Advances in sensor technology have allowed the development of “smart” sensors that are not only able to collect but also process data [1]. In addition to the sensing and processing units, these sensors have communication units that provide additional capability of dissemination of data and information. Modern sensors are inexpensive and can be deployed in large numbers to form a sensor network so that the sensors collaborate in collecting, analyzing and processing information [1]. Deployment of large sensors in a network has the benefit of increasing the resolutions of the data collected and, as a result, increasing the accuracy of the processed information. Moreover, sensor networks, by virtue of the multiplicity of the data collected from a large number of sensors, provide protections against sensor failures, mitigates the effect of shadowing due to no line-of-sight (LOS) transmission path and enhances reliability against low Signal-power-to-Noise-Ratio (SNR).

The most important feature of sensor networks is their capability to process data collaboratively. Based on their data processing architecture, sensor networks can be classified as centralized, distributed and hierarchical. In a centralized architecture, all of the data processing functions are accomplished at a central location known as a fusion center where the data is processed and information is extracted. Since the raw data from all the sensors is transmitted to the fusion center, the centralized architecture requires a huge communication bandwidth [2]. Moreover, the centralized architecture is less attractive because it is not scalable, and it is prone to the robustness of the fusion center.

The distributed architecture has no central processing unit, and, thus, the sensors locally process the collected data with limited collaboration from other sensors, typically with neighboring sensors. Since each sensor communicates only with its neighboring sensors, frequency reuse can be implemented to save bandwidth. Furthermore, transmission power is saved because the distance of signal transmission is limited to neighboring sensors. Distributed architecture has additional benefits such as reduced computational load and enhanced robustness to failures. However, distributed architecture requires more sophisticated sensor nodes, which have the capability to process data, as well as algorithms that can extract optimal information with minimal exchange of data among the sensors. This paper focuses on developing a distributed algorithm that reduces sensor communications with the objective of reducing bandwidth and transmission power.

An important application of sensor networks is for target tracking [3,4,5,6]. From a signal processing perspective, target tracking can be considered as a filtering problem [7,8]. The kinematic parameters of the target, which evolve in time, are modeled as the states of a dynamic system. The data collected from the sensors, which are functions of the kinematics parameters, represent the measurements of the dynamic system. The objective of the target tracking problem is then the estimation of the kinematic parameters, which typically include the position, velocity and acceleration of the target, recursively in real-time.

When the target tracking model is formulated as a linear and a Gaussian dynamic system, the tracking problem is analytically tractable and its solution can be obtained using a Kalman filter. However, most target tracking problems are modeled as nonlinear dynamic systems, primarily because the relationship of the measurements obtained from the sensors and the kinematic parameters is usually nonlinear [7,9]. For nonlinear dynamic systems, exact analytic solutions are generally not available; however, approximate and suboptimal solutions can be obtained using grid-based filters or extended Kalman filters (EKF) and their variants. A more accurate and popular estimation method for nonlinear and non-Gaussian dynamic systems, however, are Sequential Monte Carlo (SMC) methods or, as widely refereed, particle filtering methods [10,11].

Particle filtering methods are Bayesian methods that recursively approximate the posterior distribution of the unknown kinematic parameters using a discrete measure with a random support [12,13]. The discrete measure is defined using a set of samples, also refereed particles, and their associated weights. The particles are sequentially drawn from an importance function through Monte Carlo technique, and the weights are recursively computed using the prior and the likelihood function of the kinematic parameters.

Particle filtering methods can be implemented in a distributed manner on sensor networks [3,4,14,15]. In such implementation, each of the sensors on the network locally runs a particle filtering algorithm and exchanges information with the other sensors to approximate the global posterior distribution of the kinematic parameters. Sensor networks, however, are resource constrained, and have limited power and communication bandwidth. When the number of sensors in the network is large, the required bandwidth for communication between the sensors can be significant [16]. Moreover, in most cases, sensors consume more power for transmission of data than for data processing. These constraints limit the amount of information that can be exchanged among sensors. A critical issue in distributed particle filtering, therefore, is the development of algorithms that efficiently represent the information that is exchanged among the sensors to build the global posterior density. The objective of this paper is to develop such an algorithm.

### State-of-the-Art

There have been many distributed particle filtering (DPF) methods proposed in the literature. These distributed particle filtering algorithms can be broadly categorized into two classes in terms of the type of information they exchange among each other. The first class of DPF methods requires the exchange, among the sensors, some form of the raw measurement collected by each sensor. Recently, an example of such DPF method that requires the availability of all the raw measurements at every sensor node is proposed in [3]. This approach allows each sensor to compute the global posterior distribution locally. While such an approach provides an optimal approximation of the global posterior density, and hence, achieves excellent performance, it is, however, resource prohibitive due to its exorbitant power and bandwidth demand [17]. The second class of DPF methods requires the exchange of some statistics of the local estimate, rather than the raw measurement, of each sensor. Since the estimates represent processed information, this approach may demand lower processing load from the sensors than the methods of the first class. One such approach is described in [18], where each sensor transmits its computed statistics of the local posterior density to a central processing unit where the data is fused and the global posterior density is determined. Although the computational load of the fusion center may be reduced, due to partial data processing by the sensors, this strategy not only is not entirely distributed but also demands high power requirements because of the need for sensors located far away to send their information to the fusion center. An efficient approach to reducing the power and bandwidth requirement of a sensor network is, therefore, to develop a distributed algorithm whose communication strategy requires the sensors to exchange their statistical information to neighbouring sensors only rather than to a far-located fusion center or to all sensors. We note that the communication power requirement of sensors increases proportional at least to the square of the transmission distance. Moreover, when the sensors are restricted to exchanging information with only their neighbours, the communication bandwidth can be reused in other sensor neighbourhoods. Most of the second class of DPF methods use neighbourhood information exchange strategies such as forward-backward propagation [19] or a consensus filter [19,20,21] to compute the statistics of the global posterior distribution. The performance as well as the efficiency, in terms of power and bandwidth, of the second class of the DPF methods depend on the type and the form of the information the sensors exchange. For example, in [22] and also in [20], each sensor approximates the local posterior distribution using a Gaussian function and transmits to its neighbours the mean and covariance of the approximation to cooperatively build the global posterior distribution through a gossip (consensus) algorithm. Similarly, [23], builds the global posterior distribution from the Gaussian approximation of a local posterior through an iterative protocol based on covariance intersection. At the cost of power and bandwidth efficiency, a better approximation of the global posterior distribution can be obtained by approximating the local posterior distributions by Gaussian mixture models (GMM) as proposed in [14,24]. A DPF method where sensors collaborate to compute the global likelihood function, rather than the global posterior density, through a consensus filter has been reported [25,26,27]. The work in [25] provides a generalized approach of approximating the global likelihood through consensus filter. It approximates the log-likelihood by a polynomial function, and the sensors exchange only the coefficients of the polynomial function to compute the global likelihood.

This paper follows the approach where the sensors approximate their local likelihood functions by a Gaussian function, and, collaboratively with neighboring sensors, build the global likelihood function by exchanging the mean and covariance of their approximations. Our algorithm has similar computational cost and communication demand as the DPF methods that approximate either the likelihood or the posterior distribution by a Gaussian function. In our algorithm, the Gaussian approximated likelihood function is computed using the Monte Carlo technique. Such approximation of the likelihood function, with the availability of the prior distribution, provides better performance, particularly, for the common case where the measurement noise is Gaussian and the resulting likelihood function is uni-modal.

Our algorithm is based on the observation that, in particle filtering algorithms, the contribution of new measurements from the sensors to the recursive updating of the global posterior distribution is entirely contained within the global likelihood function. Assuming the measurement noise across all the sensors is identically and independently distributed (i.i.d.), it can be shown that the global likelihood function is a product of all the local likelihood functions. With an approximated Gaussian local likelihood functions, the global likelihood function is also a Gaussian function whose mean and covariance can be computed either by propagating the parameters of the local likelihood functions across the sensors using a forward-backward propagation strategy or by using an average consensus filter. In this paper, we have developed an algorithm that is flexible to employ both the forward-backward propagation and the average consensus filter information exchange strategies to compute the global likelihood function.

The rest of the paper is structured as follows. We present the system model in Section 2. A brief overview of the particle filtering method is given in Section 3. The proposed distributed particle filtering algorithm is laid out in Section 4. Section 5 describes the proposed strategy for information exchange among the sensors. Computer simulation results are given in Section 6, and finally conclusions are provided in Section 7.

## 2. System Model

Suppose a sensor network consisting of *K* sensors is used for tracking a target. The problem of target tracking involves the sequential estimation of the kinematic parameters of the target from the measurements obtained from the sensors. From a signal processing perspective, target tracking is a filtering problem [7,8]. The time-evolution of the kinematic parameters and the relationship of the measurements to the kinematic parameters can be modeled by a dynamic system with a state-space representation in which the kinematic parameters represent the unknown states, and the measurements obtained from the sensors represent the observations of the dynamic system. In our mathematical representation, we denote these unknown states of the dynamic system at time *n* by a vector $x_{n}={\left[x_{1,n},x_{2,n},\ldots ,x_{M_{x},n}\right]}^{⊤}$ where n=1,2,… and $M_{x}$ is the dimension of x.

Assuming the evolution of the vector $x_{n}$ has a Markovian property, we represent the state equation of the dynamic system as follows:

(1) $$x_{n}=f\left(x_{n-1}\right)+u_{n},$$

where f(·) denotes the function that describes the time-evolution of the vector $x_{n}$, and $u_{n}$ is an $M_{x}\times 1$ vector denoting the state noise. We assume the state function f(·) is nonlinear. Because of the Markovian property, the state transition probability density function (pdf), $p(x_{n}|x_{n-1})$ is solely dependent upon the pdf of $u_{n}$. Furthermore, we assume that $p(x_{n}|x_{n-1})$ is known.

We represent the observation equation of the dynamic system as follows:

(2) $$y_{k,n}=h_{k}\left(x_{n}\right)+e_{k,n}\;for\;k=1,\ldots ,K,$$

where $y_{k,n}$ is a vector of dimension $M_{y}$ and denotes the measurement obtained from sensor *k* at time instant *n*; $h_{k}\left(\cdot \right)$ denotes the measurement function that maps the kinematic vector parameter $x_{n}$ to the measurement vector $y_{k,n}$; and $e_{k,n}$ is a vector of dimension $M_{y}$ denoting the measurement noise. We assume that the measurement function $h_{k}\left(\cdot \right)$ is nonlinear but known. Furthermore, we assume that the pdf of the measurement noise vector $e_{k,n}$ is known, and it is identically and independently distributed (i.i.d.) across time and sensors. Therefore, the local likelihood function of each sensor $p(y_{k,n}|x_{n})$, for k=1,…,K are known.

From the above formulation, the objective of the tracking problem is the sequential real-time estimation of the state vectors $x_{0:n}$ from the measurements $Y_{1:n}$, where $x_{0:n}=\left\{x_{0},x_{1},\ldots ,x_{n}\right\}$ denotes the values of the states from time 0 to time *n*, and $Y_{1:n}=\left\{y_{1:n,1},y_{1:n,2},\ldots ,y_{1:n,K}\right\}$ denotes the measurements obtained from all the sensors from time 1 to time *n*.

## 3. Particle Filtering Method for Sensor Networks

When a dynamic system represented by Equations (1) and (2) is nonlinear and/or non-Gaussian, there is no analytical solution for the sequential estimation of $x_{0:n}$ from the measurement $Y_{1:n}$. In such a case, we resort to numerical methods for approximate solutions, and one such powerful method is particle filtering. Particle filtering is a Monte Carlo technique whose estimation principles are rooted in Bayesian methodology. According to Bayesian methodology, all the information needed to estimate the unknown parameter $x_{0:n}$ is captured by its posterior distribution, $p(x_{0:n}|Y_{1:n})$ [12,13].

Recently, many variants of particle filtering methods have been proposed [28,29,30]. However, in its basic form, particle filtering recursively estimates the posterior pdf $p(x_{0:n}|Y_{1:n})$ by approximating it using a random discrete measure defined as $X_{n}={\left\{x_{0:n}^{\left(i\right)},w_{n}^{\left(i\right)}\right\}}_{i=1}^{N}$, where $x_{0:n}^{\left(i\right)}$ is the *i*-th random particle trajectory, $w_{n}^{\left(i\right)}$ is its corresponding weight and *N* is the total number of particles [13]. Mathematically, the approximate posterior pdf is represented by

(3) $$\hat{p}\left(x_{0:n}|Y_{1:n}\right)=\sum_{i=1}^{N}\delta \left(x_{0:n}-x_{0:n}^{\left(i\right)}\right)w_{n}^{\left(i\right)},$$

where δ(·) is the Dirac’s delta function.

A key concept in the development of particle filtering is importance sampling [13]. According to importance sampling, an empirical approximation of the posterior distribution $p(x_{0:n}|Y_{1:n})$ can be obtained using samples drawn from an importance function $\pi (x_{0:n}|Y_{1:n})$ by attaching corresponding weights that are computed from

(4) $$w_{n}^{\left(i\right)}=\frac{p(x_{0:n}^{\left(i\right)}|Y_{1:n})}{\pi (x_{0:n}^{\left(i\right)}|Y_{1:n})}.$$

A critical feature of particle filtering technique is the ability to recursively build the random discrete measure at time *n* from the discrete measure obtained at time n-1. When new information of $Y_{n}$ is received at time *n*, the discrete measure $X_{n}$ of posterior distribution $p(x_{0:n}|Y_{1:n})$ is built from the discrete measure $X_{n-1}$ of $p(x_{0:n-1}|Y_{1:n-1})$ by simply appending the new particles $x_{n}^{\left(i\right)}$ and updating the weight $w_{n}^{\left(i\right)}$.

The recursive approximation of the posterior distribution requires an importance function that can be factorized as follows [10]:

(5) $$\pi \left(x_{0:n}|Y_{1:n}\right)=\pi \left(x_{n}|x_{0:n-1},Y_{1:n}\right)\pi \left(x_{0:n-1}|Y_{1:n-1}\right).$$

With such choice of an importance function, it can be shown that the weights update equation is given by

(6) $$w_{n}^{\left(i\right)}\propto w_{n-1}^{\left(i\right)}\frac{p\left(Y_{n}|x_{n}^{\left(i\right)}\right)p\left(x_{n}^{\left(i\right)}|x_{n-1}^{\left(i\right)}\right)}{\pi (x_{n}|x_{0:n-1},Y_{1:n})}.$$

It is shown that, as the number of particles goes to infinity, the approximated posterior distribution converges to the true posterior distribution [11]. After approximating the posterior distribution by its discrete measure, then a Minimum Mean Square Estimator (MMSE) given by

(7) $$x_{n}^{mmse}=\sum_{i=1}^{N}x_{n}^{\left(i\right)}w_{n}^{\left(i\right)}$$

can be applied to compute the estimates of $x_{n}$.

If the particle filtering algorithm is implemented as described above, the particles would degenerate after a few iterations, i.e., most of the particles except for a very few would have insignificant weights [13]. Because the particles with small weights have little contribution to the estimation of the unknown parameters, carrying them forward is simply a waste of computational resources. Moreover, it has been reported that the variance of the weights of the particles does not decrease as the iteration continues [13]. A common approach to avoid such particle degeneracy is to apply resampling [31], which is a procedure that essentially replicates the particles with significant weights while eliminating those particles with very small weights. Resampling is a Monte Carlo operation where the particles that are carried forward to time *n* are stochastically chosen from the particles at time n-1 with a probability proportional to their weights [32].

## 4. Distributed Particle Filter

To develop a distributed particle filtering algorithm in which multiple sensors collaborate to estimate the unknown states, let us consider the following decomposition of the posterior distribution

(8) $$\begin{array}{cc} p(x_{0:n}|Y_{0:n}) & \propto p\left(Y_{n}|x_{0:n},Y_{0:n-1}\right)p\left(x_{0:n}|Y_{0:n-1}\right)\\ & \propto p\left(Y_{n}|x_{n}\right)p\left(x_{n}|x_{n-1}\right)\times p\left(x_{0:n-1}|Y_{0:n-1}\right)\\ & =\prod_{k=1}^{K}p\left(y_{n,k}|x_{n}\right)p\left(x_{n}|x_{n-1}\right)\times p\left(x_{0:n-1}|Y_{0:n-1}\right), \end{array}$$

which is derived using Baye’s theorem by assuming Markovian relationship of the states and independent noise measurements across sensors and time. Selecting the prior pdf (predictive density) of the state variable as the importance function,

$$\pi (x_{n}|x_{0:n-1},y_{1:n})=p\left(x_{n}^{\left(i\right)}|x_{n-1}^{\left(i\right)}\right),$$

the weight update equation is obtained as follows:

(9) $$w_{n}^{\left(i\right)}\propto w_{n-1}^{\left(i\right)}\prod_{k=1}^{K}p\left(y_{n,k}|x_{n}^{\left(i\right)}\right),$$

where $p(y_{n,k}|x_{n})$ represents the local likelihood function of sensor *k*, and the product $\prod_{k=1}^{K}p\left(y_{n,k}|x_{n}\right)$ denotes the global likelihood function.

In the estimation of the global posterior distribution, we note from the weight update Equation (9) that all the measurement information from the sensors is captured by the global likelihood function. Therefore, if a sensor can somehow determine the global likelihood function at every instant of *n*, then it can make a local estimation of the global posterior distribution and, as a result, estimate the unknown state $x_{n}$ recursively. Furthermore, we note that the global likelihood function is a product of the local likelihood function of all the sensors. In building the estimates of the posterior distribution, the sensors, therefore, need to exchange information about their local likelihood function to determine the global likelihood function. It is shown that the distributed particle filters uniformly convergence [15].

### Proposed Algorithm

In our proposed algorithm, at each instant of time *n*, when a sensor *k* obtains a measurement $Y_{n,k}$, it approximates its local likelihood function, $p(y_{n,k}=Y_{n,k}|x_{n})$, by a Gaussian function given by

(10) $$p\left(y_{n,k}=Y_{n,k}|x_{n}\right)\approx N_{n,k}\left(x;\mu_{n,k},\Sigma_{n,k}\right),$$

where $N_{n,k}\left(\cdot \right)$ denotes the Gaussian function, $\mu_{n,k}$ is the mean, and $\Sigma_{n,k}$ is the covariance of the Gaussian function.

The approximation of the local likelihood function by a Gaussian function is performed using Monte Carlo techniques. The statistics of the approximated Gaussian function are determined from the sample mean, $\mu_{n,k}$, and sample covariance, $\Sigma_{n,k}$, computed from particles obtained from the predictive density. Since the importance function for our particle filtering algorithm is chosen to be the predictive density, better approximation of the local likelihood function can be obtained if particles, ${\left\{x_{n}^{\left(i\right)}\right\}}_{i=1}^{N}$, obtained from the predictive density, are used for the Monte Carlo computation of the sample mean and sample covariance of the approximated Gaussian function.

The computation of the sample mean and covariance of the Gaussian function require the determination of a likelihood factor ${\tilde{\alpha }}_{i,k}$, which is obtained by evaluating the value of $p(y_{n,k}=Y_{n,k}|x_{n})$ at $x_{n}=x_{n}^{\left(i\right)}$, where $x_{n}^{\left(i\right)}$ are particles drawn from $p(x_{n}^{\left(i\right)}|x_{n-1}^{\left(i\right)})$, i.e.,

(11) $${\tilde{\alpha }}_{i,k}=p\left(y_{n,k}=Y_{n,k}|x_{n}\right){|}_{x_{n}=x_{n}^{\left(i\right)}}.$$

After determining the likelihood factors, the sample mean $\mu_{n,k}$ is determined from

(12) $$\mu_{n,k}=\sum_{i=1}^{N}\alpha_{i,k}x_{n}^{\left(i\right)},$$

and the sample covariance $\Sigma_{n,k}$ is computed from

(13) $$\Sigma_{n,k}=\sum_{i=1}^{N}\alpha_{i,k}\left(x_{n}^{\left(i\right)}-\mu_{n,k}\right){\left(x_{n}^{\left(i\right)}-\mu_{n,k}\right)}^{⊤},$$

where $\alpha_{i,k}=\frac{{\tilde{\alpha }}_{i,k}}{\sum_{j=1}^{N}{\tilde{\alpha }}_{j,k}}$ is the normalized likelihood factor of the *i*-th particle of the *k*-th sensor.

Since the local likelihood function of each sensor is approximated by a Gaussian function, the global likelihood function, which is the product of all the local likelihood functions, is also a Gaussian function. The approximate global likelihood function is then given by

(14) $$\begin{array}{ccc} \prod_{k=1}^{K}p\left(y_{n,k}=Y_{n,k}|x_{n}\right) & \approx & \prod_{k=1}^{K}N_{n,k}\left(x;\mu_{n,k},\Sigma_{n,k}\right)\\ & = & N(x;{\bar{\mu }}_{n},{\bar{\Sigma }}_{n}), \end{array}$$

where ${\bar{\mu }}_{n}$ is the global mean and ${\bar{\Sigma }}_{n}$ is the global covariance. It is straightforward to show that the global covariance can be computed from the covariance of the local likelihood functions by

(15) $${\bar{\Sigma }}_{n}^{-1}=\sum_{k=1}^{K}\Sigma_{n,k}^{-1},$$

and the global mean can be determined using

(16) $${\bar{\mu }}_{n}{\bar{\Sigma }}_{n}^{-1}=\sum_{k=1}^{K}\mu_{n,k}\Sigma_{n,k}^{-1}.$$

From Equations (15) and (16), we observe that the computation of the global covariance and the product of mean and covariance entails only a simple summation of local parameters, which makes our algorithm convenient to apply the forward-backward propagation and average consensus filter strategies for information exchange among the sensors.

## 5. Strategy for Information Exchange

After computing the mean and covariance of their local likelihood functions using Equations (12) and (13), respectively, the sensors exchange these values to compute collaboratively the mean and covariance of the global likelihood functions using Equations (15) and (16). If each sensor attempts to broadcast its values to all the sensors, the required transmission power and bandwidth could be extremely high. The power and the bandwidth required for the transmission of information among the sensors will be limited if the sensors are restricted to communicate with their neighbours. We note that when a signal is transmitted over a wireless channel from a source, its received power decreases inversely proportional to at least the square of the transmission distance. Thus, the amount of power required to reach the far located sensors is much greater than the power required to transmit information to neighbouring sensors. Furthermore, when transmission of information is limited to neighbouring sensors, bandwidth can be reused in other sensor neighbourhoods, saving the total bandwidth required for the sensor networks.

The simplest strategy for exchanging information among the sensors is the forward-backward propagation strategy in which each sensor passes on information on a path exactly once in the forward and then once in the backward directions. In its simplest form, the forward-backward strategy, therefore, first requires the determination of a path that traverses through all the sensors once by connecting each sensor to one of its neighbors. In the forward direction, the data of each sensor is accumulated and the total sum is obtained at the last sensor, then the total sum is propagated backwards to each sensor through the same pre-determined path.

For our algorithm, the forward propagation involves the following steps:

- *the sensor at node j receives partial sums* $\sum_{k=1}^{j-1}\Sigma_{n,k}^{-1}$ *and* $\sum_{k=1}^{j-1}\mu_{n,k}\Sigma_{n,k}^{-1}$ *from sensor at node* j-1
- *adds its computed local values,* $\Sigma_{n,j}^{-1}$ *and* $\mu_{n,j}\Sigma_{n,j}^{-1}$ *to the partial sums*
- *produces its partial sums,* $\sum_{k=1}^{j}\Sigma_{n,k}^{-1}$ *and* $\sum_{k=1}^{j}\mu_{n,k}\Sigma_{n,k}^{-1}$*, and transmits them to sensor node* j+1

After the forward propagation is completed, the values of the global covariance and the product of the mean and covariance are propagated backwards to each sensor through the established direct path. The complete algorithm of the forward-backward information exchange strategy is given in Algorithm 1.

**Algorithm 1** Forward Backward Exchange1. *Forward Propagation*For j=2
*to K* (the path is pre-determined)$\sum_{k=1}^{j}\Sigma_{n,k}^{-1}=\Sigma_{n,j}^{-1}+\sum_{k=1}^{j-1}\Sigma_{n,k}^{-1}$$\sum_{k=1}^{j}\mu_{n,k}\Sigma_{n,k}^{-1}=\mu_{n,j}\Sigma_{n,j}^{-1}+\sum_{k=1}^{j-1}\mu_{n,k}\Sigma_{n,k}^{-1}$*end*2. Backward propagation*For*
j=K:-1:1 (backward)*Pass*
$\sum_{k=1}^{K}\Sigma_{n,k}^{-1}$ to sensor at node *j**Pass*
$\sum_{k=1}^{K}\mu_{n,k}\Sigma_{n,k}^{-1}$ to sensor at node *j**end*

While simplicity and pre-determined latency are the two major advantages of the forward-backward propagation strategy, it also has important drawbacks. First of all, the latency is directly proportional to the number of sensors, and if the sensor network has a large number of sensors, the latency could be huge. The second drawback is that the strategy requires the pre-determination of a direct path that traverses through all the sensors, and whenever a sensor fails, the path should be updated [19]. Since the information exchange in our algorithm requires a simple accumulation of values, the latency problem can be alleviated by using multiple paths that converge at single or multiple sensors.

An alternative strategy of information exchange that limits communication with neighboring sensors is the use of an average consensus filter. An average consensus algorithm is an iterative procedure for computing an average of the values held by a network of nodes [4]. While the exchange of values is limited to neighbouring nodes, the algorithms converge asymptotically to a global agreement. See [33] for more discussion on convergence of consensus filters. In our case, the sensors compute iteratively the values of the average of the inverse of the covariance and the average of the product of the mean and the inverse of the covariance by exchanging their computed values only with their neighbours.

The steps of the average consensus filter for our algorithm are as follows. Suppose that at iteration m-1, sensor *j* receives from all its single-hop neighbors, $k=1,\ldots ,N_{j}$, the averages, ${\left\{{\left({\bar{\Sigma }}^{-1}\right)}_{n,k}^{\left(ave\right)}\left(m-1\right)\right\}}_{k=1}^{N_{j}}$ and ${\left\{{\left(\bar{\mu }{\bar{\Sigma }}^{-1}\right)}_{n,k}^{-1}\left(m-1\right)\right\}}_{k=1}^{N_{j}}$, where $N_{j}$ denotes the total number of single-hop neighbors of sensor *j*. Then, sensor *j* updates the averages at iteration *m* as follows:

(17) $$\begin{array}{ccc} {\left(\bar{\mu }{\bar{\Sigma }}^{-1}\right)}_{n,j}^{\left(ave\right)}\left(m\right) & = & \epsilon \sum_{k=1}^{N_{j}}{\left(\bar{\mu }{\bar{\Sigma }}^{-1}\right)}_{n,k}^{\left(ave\right)}\left(m-1\right)-{\left(\bar{\mu }{\bar{\Sigma }}^{-1}\right)}_{n,j}^{\left(ave\right)}\left(m-1\right)\\ {\bar{\Sigma }}_{n,j}^{-1(ave)}\left(m\right) & = & \epsilon \sum_{k=1}^{N_{j}}\left({\bar{\Sigma }}_{n,k}^{-1(ave)}\left(m-1\right)-{\bar{\Sigma }}_{n,j}^{-1(ave)}\left(m-1\right)\right) \end{array}$$

where *ϵ* is a constant step size. It is known that if *ϵ* is small and k→∞, the iteration converges [34] to ${\bar{\Sigma }}_{n}^{-1(ave)}=\frac{1}{K}\sum_{k=1}^{K}{\bar{\Sigma }}_{n,k}^{-1}$ and ${\bar{\mu }}_{n}{\bar{\Sigma }}_{n}^{-1(ave)}=\frac{1}{K}\sum_{k=1}^{K}{\bar{\mu }}_{n,k}{\bar{\Sigma }}_{n,k}^{-1}$, from which the desired ${\bar{\Sigma }}_{n}^{-1}$ and ${\bar{\mu }}_{n}{\bar{\Sigma }}_{n}^{-1}$ can be computed. The algorithm is summarized in Algorithm 2.

**Algorithm 2** Average Consensus Algorithm1. *Initializing for every sensor j- iteration zero*${\bar{\Sigma }}_{n,j}^{-1(ave)}\left(0\right)={\bar{\Sigma }}_{n,j}^{-1}$${\left(\bar{\mu }{\bar{\Sigma }}^{-1}\right)}_{n,j}^{\left(ave\right)}\left(0\right)={\left(\bar{\mu }{\bar{\Sigma }}^{-1}\right)}_{n,j}$2. Iterate until convergencewhile(NotConv) *(*Δ *is a small convergence test constant)*m=m+1 (increment iteration index)3. Repeat for all sensors *j*${\left(\bar{\mu }{\bar{\Sigma }}^{-1}\right)}_{n,j}^{\left(ave\right)}\left(m\right)=\epsilon \sum_{k=1}^{N_{j}}{\left(\bar{\mu }{\bar{\Sigma }}^{-1}\right)}_{n,k}^{\left(ave\right)}\left(m-1\right)-{\left(\bar{\mu }{\bar{\Sigma }}^{-1}\right)}_{n,j}^{\left(ave\right)}\left(m-1\right)$${\bar{\Sigma }}_{n,j}^{-1(ave)}\left(m\right)=\epsilon \sum_{k=1}^{N_{j}}\left({\bar{\Sigma }}_{n,k}^{-1(ave)}\left(m-1\right)-{\bar{\Sigma }}_{n,j}^{-1(ave)}\left(m-1\right)\right)$$Conv=|{\left(\bar{\mu }{\bar{\Sigma }}^{-1}\right)}_{n,j}^{\left(ave\right)}\left(m\right)-{\left(\bar{\mu }{\bar{\Sigma }}^{-1}\right)}_{n,j}^{\left(ave\right)}\left(m-1\right)|<\Delta \;\&\&$$|{\bar{\Sigma }}_{n,j}^{-1(ave)}\left(m\right)-{\bar{\Sigma }}_{n,j}^{-1(ave)}\left(m-1\right)|<\Delta$*end*

The summary of the steps of the proposed distributed particle filtering algorithm is given in Algorithm 3.

**Algorithm 3** Distributed particle filtering Algorithm1.   Each Sensor *k* performs the following:2.   Initializing        All sensor measurements are used to derive priors3.    For n=3 to $N_{T}$
*(total time instants)*4.       *For*
i=1
*to N* (total number of particles)5.          Drawing a sample $x_{n}^{\left(i\right)}$             $x_{n}^{\left(i\right)}\propto p\left(x_{n}|x_{n-1}^{\left(i\right)}\right)$6.          Computing the likelihood factors       ${\tilde{\alpha }}_{i,k}=p\left(y_{n,k}=Y_{n,k}|x_{n}\right){|}_{x_{n}=x_{n}^{\left(i\right)}}$    and $\alpha_{i,k}=\frac{{\tilde{\alpha }}_{i,k}}{\sum_{j=1}^{N}{\tilde{\alpha }}_{j,k}}$7.          Computing the local mean and Covariance              $\mu_{n,k}=\sum_{i=1}^{N}\alpha_{i,k}x_{n}^{\left(i\right)}$              $\Sigma_{n,k}=\sum_{i=1}^{N}\alpha_{i,k}\left(x_{n}^{\left(i\right)}-\mu_{n,k}\right){\left(x_{n}^{\left(i\right)}-\mu_{n,k}\right)}^{⊤}$8.       Approximating the local likelihood function          $p\left(y_{n,k}=Y_{n,k}|x_{n}\right)\approx N_{n,k}\left(x;\mu_{n,k},\Sigma_{n,k}\right)$9.       Computing global likelihood function          (by exchanging information with other sensors)            $\prod_{k=1}^{K}p\left(y_{n,k}=Y_{n,k}|x_{n}\right)\approx N\left(x;{\bar{\mu }}_{n},{\bar{\Sigma }}_{n}\right)$            where  ${\bar{\Sigma }}_{n}^{-1}=\sum_{k=1}^{K}\Sigma_{n,k}^{-1}$  and ${\bar{\mu }}_{n}{\bar{\Sigma }}_{n}^{-1}=\sum_{k=1}^{K}\mu_{n,k}\Sigma_{n,k}^{-1}$10.        Weight Updating $w_{n}^{\left(i\right)}$              ${\tilde{w}}_{n}^{\left(i\right)}\propto w_{n-1}^{\left(i\right)}N\left(x_{n}^{\left(i\right)};{\bar{\mu }}_{n},{\bar{\Sigma }}_{n}\right)$11.       Weight Normalization $w_{n}^{\left(i\right)}={\left(\sum_{n=1}^{N}{\tilde{w}}_{n}^{\left(n\right)}\right)}^{-1}{\tilde{w}}_{k}^{\left(i\right)}$12.       Resampling if $N_{eff}=\frac{1}{\Sigma_{i=1}^{N}{\left(w_{n}^{\left(i\right)}\right)}^{2}}<N/2$    $x_{n}^{mmse}=\sum_{i=1}^{N}x_{n}^{\left(i\right)}w_{n}^{\left(i\right)}$

## 6. Simulation Results

**Figure 1:**
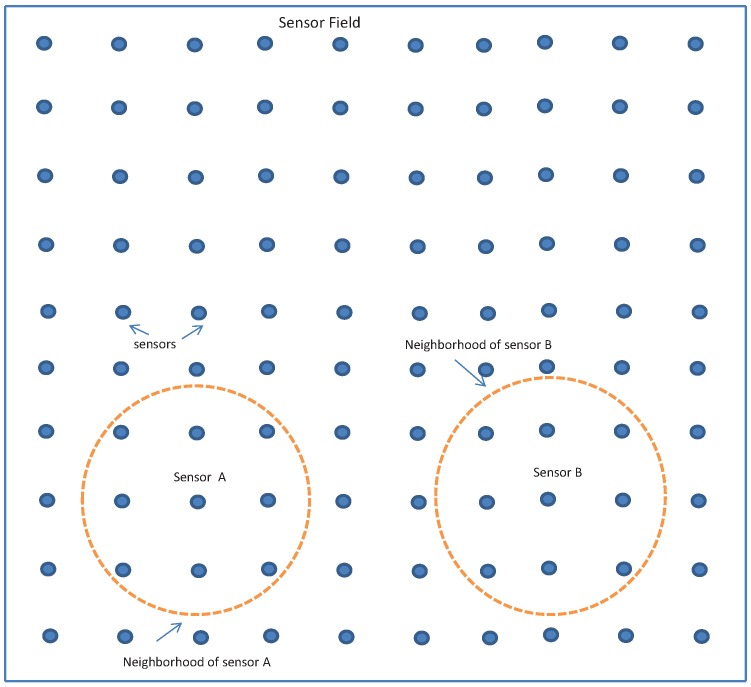
Sensor network layout.

A computer simulation was carried out to verify the effectiveness of the proposed algorithm. A target tracking problem on a two-dimensional plane (*x*–*y*) was considered. A sensor network consisting of 100 sensors laid out on a 200 m × 200 m field, as shown in Figure 1, is used to collect and process the target tracking data.

The motion of the target is assumed to be constant velocity and the evolution of its kinematic parameters are modeled by

(18) $$\begin{array}{c} x_{n}=Fx_{n-1}+Gu_{n}, \end{array}$$

where the state vector denoted by $x_{n}={\left[x_{n}\;y_{n}\;{\dot{x}}_{n}\;{\dot{y}}_{n}\right]}^{⊤}$ consists of the *x*–*y* positions ($x_{n}$ and $y_{n}$) and velocities (${\dot{x}}_{n}$ and ${\dot{y}}_{n}$) of the target,

$$F=\left[\begin{array}{cccc} 1 & 0 & T_{s} & 0\\ 0 & 1 & 0 & T_{s}\\ 0 & 0 & 1 & 0\\ 0 & 0 & 0 & 1 \end{array}\right],G=\left[\begin{array}{cc} \frac{T_{s}^{2}}{2} & 0\\ 0 & \frac{T_{s}^{2}}{2}\\ Ts & 0\\ 0 & Ts \end{array}\right].$$

$T_{s}$ is the sampling period (we assume $T_{s}=1$), and $u_{n}∼N\left(0,\Sigma_{u}\right)$ is a two-dimensional Gaussian noise vector with a covariance of $\Sigma_{u}=\mathrm{diag}\left\{0.005,0.005\right\}$.

The measurements obtained by the sensors are expressed as a function of the distance of the sensors from the location of the target is given by the measurement equation

(19) $$\begin{array}{ccc} z_{k,n} & = & \frac{C}{{\left(x_{n}-s_{x,k}\right)}^{2}+{\left(y_{n}-s_{y,k}\right)}^{2}}+e_{k,n}, \end{array}$$

where $z_{k,n}$ denotes the measured signal at time *n* by sensor *k*, $s_{x,k}$ and $s_{y,k}$ denote the *x*- and *y*-axis location of sensor *k*, C=570 is a constant chosen to calibrate the SNR of the signal received by the furthest sensor to be 10dB when the target is located at the center, and $e_{k,n}∼N\left(0,\sigma_{e}^{2}\right)$ is a measurement of Gaussian noise of sensor *k* with a unity variance, $\sigma_{e}^{2}=1$.

In the first experiment, we run simulations of the proposed distributed particle filtering algorithm using the forward-backward propagation and the average consensus filtering information exchange strategies for the sensors to collaboratively compute the global likelihood function. In this experiment, the consensus filters were run for seven iterations, and we assumed that the neighbors of a sensor include only the sensors that are immediately adjacent in the vertical, horizontal and diagonal directions. For both strategies, the distributed particle filtering algorithm that was run at each local sensor node used a particle size of 500. For comparison, as a lower bound, we have also conducted an experiment where the sensors transmit their raw measurements to a fusion center, which computes the exact global likelihood function to estimate the position of the target.

We note that for distanced-based measurement equations such as Equation (19), the likelihood is a circularly-symmetric or ring-shaped function, i.e., all particles that are equidistant from any sensor location produce the same local likelihood function. Therefore, for this specific measurement equation, our algorithm requires an informative prior distribution. There are several ways of obtaining a prior distribution; for example, it can be assumed Gaussian distributed whose mean is the estimate obtained through a triangulation method using the first few measurements of sensors selected strategically. In our simulation, however, the prior distribution was obtained with the assumption that initially the first three measurements of each sensor was available to all sensors. Each sensor applies particle filtering method using measurements from all sensors to obtain an estimate of the location of the target at the end of the third measurement. Then, the estimate of each sensor is taken as the mean of its Gaussian prior distribution. The covariance of the Gaussian prior distribution is assumed $3\Sigma_{u}$.

As a performance measure, we have computed the Root Mean Square Error (RMSE) of the estimated target position $\left({\hat{x}}_{n},{\hat{y}}_{n}\right)$ at each time instant *n* by averaging the estimates from all sensors. The RMSE is computed by

$$RMSE\left(n\right)=\sqrt{\frac{1}{R}\sum_{r=1}^{R}{\left(x_{n}^{\left(r\right)}-{\hat{x}}_{n}^{\left(r\right)}\right)}^{2}+{\left(y_{n}^{\left(r\right)}-{\hat{y}}_{n}^{\left(r\right)}\right)}^{2}},$$

where R=1000 is the number of simulation runs over which the RMSE is averaged.

**Figure 2:**
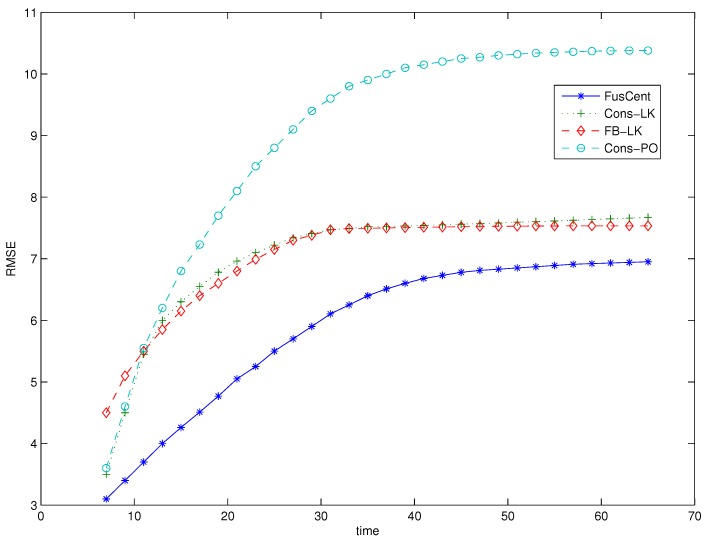
Root Mean Square Error (RMSE) versus time.

Figure 2 shows the plots of the RMSE of the estimated target position $\left({\hat{x}}_{n},{\hat{y}}_{n}\right)$ as time instant *n* evolves from n=7,…,65. In the figure, the plots denoted by FB-LK, Cons-LK and FusCent correspond to the results of the algorithms that used the information exchange strategies of forward-backward propagation, average consensus filter and fusion center, respectively. For comparison purposes, we have simulated an algorithm with comparable complexity and communication demand as our proposed algorithm. The algorithm, which is similar to [22], approximates the global posterior distribution using average consensus filter by allowing each sensor to exchange its Gaussian approximated local posterior distributions with its neighbouring sensors. This Gaussian approximated posterior algorithm used the same parameters such as the number of particles, number of consensus iterations and also all-measurement available initialization as our algorithm. The plot of the algorithm is denoted by Cons-PO.

We note from Figure 2 that the RMSE of proposed methods, FB-LK and Cons-LK, and that of the Cons-PO, initially increase with time because of the effect of the informative priors, which assume the availability of the data obtained by all the sensors at each sensor node. We observe that the RMSE of all the methods eventually level off with time as the effect of the informative priors wears off. In Figure 2, we started the plots from n=7 by ignoring the first few estimates that skewed the result favourably due to the the effect of the informative priors.

From Figure 2, we observe the proposed algorithms, both with the forward-backward propagation and average consensus filter, displayed performance that is close to that of the fusion center based algorithm and better performance than the algorithm with Gaussian approximated posterior. We emphasise, however, in comparison to our algorithms, that the fusion-center based algorithm requires much more power and bandwidth resources because the sensors need to transmit their raw measurement data to the fusion center. The total transmission power requirement of the fusion center based algorithm is proportional to the average of at least the square of the distance of the sensors to the location of the fusion center. Assuming that the fusion center is located at the center of the sensor field shown in Figure 1, the average of the square of the distance from the sensors to the fusion center is 6600, which is 16.5 times bigger than the square of the maximum distance between two sensors in the same neighbourhood. In other words, the power requirement of the fusion center architecture is 16.5 times higher than the transmission power required by our algorithm that uses a consensus filter information exchange strategy. Moreover, when using the consensus filter based algorithm, the same bandwidth can be reused by all neighborhoods while adopting a time-division multiple access (TDMA) protocol for sensors in a neighbourhood to share the bandwidth. With this scheme, the total bandwidth-time slot for the consensus algorithm is equal to the number of sensors in one neighbourhood (nine in our case) multiplied by the number of iterations (7 in our case). However, for the fusion-centered algorithm, the corresponding bandwidth-time slot grows linearly with the number of sensors. In fact, it is numerically equal to the total number of sensors in the field.

In the second experiment, we explored the effect of the number of particles on the performance of the algorithms by computing the averaged RMSE (ARMSE) of the estimated target position. The ARMSE is computed from

$$ARMSE\left(n\right)=\sqrt{\frac{1}{N_{T}R}\sum_{r=1}^{R}\sum_{n=N_{1}}^{N_{T}}{\left(x_{n}^{\left(r\right)}-{\hat{x}}_{n}^{\left(r\right)}\right)}^{2}+{\left(y_{n}^{\left(r\right)}-{\hat{y}}_{n}^{\left(r\right)}\right)}^{2}}.$$

**Figure 3:**
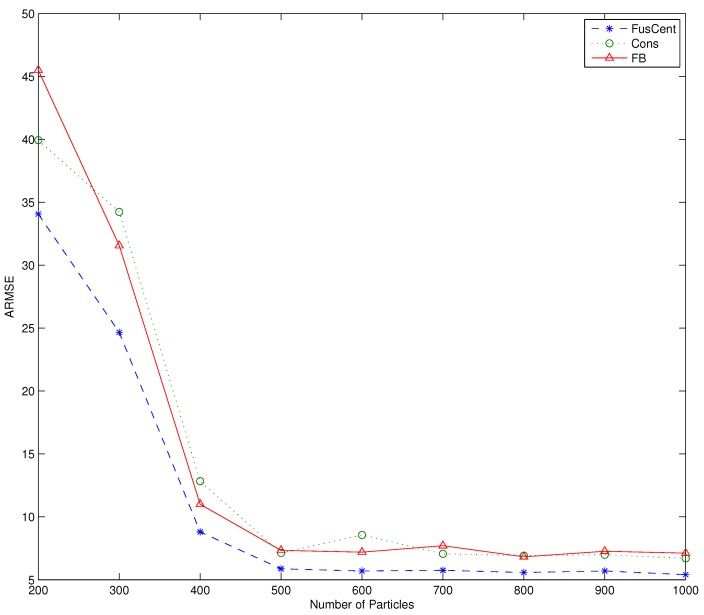
Averaged Root Mean Square Error (ARMSE) versus particle size.

Each value of of the ARMSE is averaged over the time interval of $N_{1}=7$ and $N_{T}=65$, and R=100 for the number of simulation runs. Figure 3 shows the plot of ARMSE versus the number of particles for the three algorithms. We observe from Figure 3 that the algorithms display performance improvement as the particle size is increased to N=500, but beyond N=500, the performance gain remains essentially flat. We also note that in all our simulations the performances of the forward-backward propagation and consensus filter based algorithms are practically the same.

**Figure 4:**
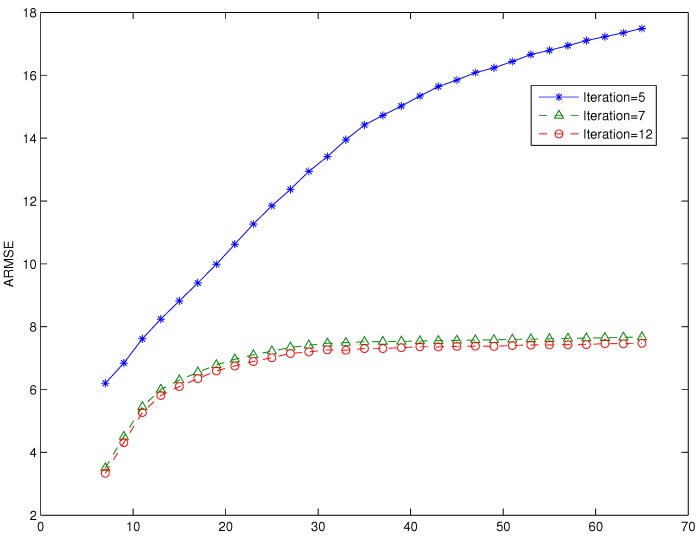
RMSE versus time for different iteration values of the consensus filter.

**Figure 5:**
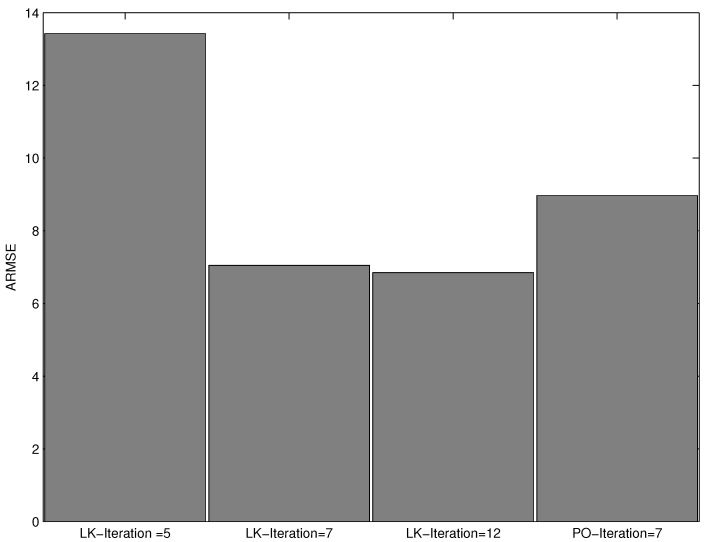
ARMSE versus number of iterations of the consensus filter.

In the third experiment, we explored the effect of varying the number of iterations of the consensus filter based algorithm. We run the consensus filter based algorithm with particle size N=500, and the number of iterations of 5, 7 and 12. The performance, in terms of RMSE, of the proposed consensus filter based algorithm as the number of iterations of the consensus filter is varied and plotted in Figure 4. A similar plot of ARMSE versus the number of iterations is shown Figure 5. In Figure 5, we have also included the plot of the ARMSE for an iteration of 12. As seen from both figures, the performance improves significantly as the number of iterations is increased from 5 to 7; however, as the number of iterations is increased from seven to 12, there is minimal performance gain. We also note that our algorithm outperforms the algorithm based on a Gaussian approximated posterior for the same number of iterations (iterations = 7). The plots denoted by LK represent the results from our proposed algorithm, while the plot denoted by PO represented the result from the algorithm based on Gaussian approximated posterior.

## 7. Conclusions

We have presented distributed target tracking algorithms for sensor networks based on a particle filtering method in which a network of sensors collaborates to estimate the kinematic parameters of the target. Our main objective in the design of the algorithms is reducing the amount of overhead data exchanged by the sensors when collaboratively estimating the parameters of interest. In a particle filtering algorithm, where the prior density is used as an importance function, the contribution of the measurement data is contained in the global likelihood function used to update the weight of the particles. Therefore, using this fact, we developed an algorithm that enables the sensors to compute the global likelihood function collaboratively by approximating their local likelihood functions by Gaussian functions using the Monte Carlo method. The approximation of the local likelihood function by Gaussian functions reduces the amount of information that needs to be exchanged among the sensors. Moreover, to reduce the power and bandwidth requirements for exchanging information, we have proposed the use of an average consensus filter or forward-backward propagation strategy that limits the information exchange of each sensor to only its neighbours. We have demonstrated the effectiveness of our algorithms through computer simulations.
